# Well-being assessment in medical students since the COVID-19 pandemic: a mixed method study

**DOI:** 10.30476/JAMP.2022.93642.1542

**Published:** 2022-04

**Authors:** SARAH MICHAUD, OUASSIM MANSOURY, LATIFA ADARMOUCH, MOHAMED AMINE, FRANCIS GUILLEMIN, MAJDA SEBBANI

**Affiliations:** 1 Clinical Research Department, Mohammed VI University Hospital, Marrakesh, Morocco; 2 Community Medicine and Public Health Department, Bioscience and Health Research Lab, School of Medicine, Cadi Ayyad University, Marrakesh, Morocco; 3 INSERM, CIC-EC CIC1433, Nancy, France; 4 Lorraine University, EA 4360 Apemac Nancy, France

**Keywords:** Medical students, Health promotion, Psychological adaptation, COVID19

## Abstract

**Introduction:**

Since the COVID-19, changes have occurred for the Moroccan medical students, which represent a vulnerable population. Coping with this situation could be difficult. Our objective was to estimate and understand the psychosocial barriers to the medical students’ well-being at the Faculty of Medicine and Pharmacy of Marrakesh (FMPM) by evaluating their coping strategies, difficulties and needs.

**Methods:**

We conducted a mixed method study among pre-graduate medical students. For the quantitative part, we did a cross-sectional study using an online four-part self-administered questionnaire. We compared Likert scales of perceived well-being before and one year after the lockdown. The scales ranged from 0 (very low state of well-being) to 10 (complete state of well-being). Coping strategies were assessed by the Brief-COPE questionnaire. The qualitative perspective was a case-study with semi-structured interviews using an interview guide based on the literature review. Finally, a one-phase triangulation analysis, underlined by a convergence model, was done.

**Results:**

We had 355 participants for the quantitative part (participation rate of 16.6%). The mean age was 19.2±1.6. The female/male sex ratio was 1.8. The first cycle students represented 76%. The well-being mean state was better before than after the pandemic (7.8 vs 5.4; p<0.001). The main coping strategy was the acceptance of the situation (5.8±1.7). According to the students, their principal need for promoting their well-being at the faculty was having courses about technologies for studies (89.3%). For the qualitative part, we interviewed 16 students. Thirteen had a decline of their well-being after the lockdown. Isolation and adaptation to e-learning were the principal difficulties. However, mainly, they adopted engaging in coping strategies.

**Conclusion:**

The medical students’ well-being decreased since the COVID-19 pandemic. Students adopting coping strategies were in the best well-being state. Psychosocial and solution-based measures should be put in place at the FMPM to foster the students’ well-being.

## Introduction

The new coronavirus, SARS-COV-2, induced a global pandemic all over the world. Countries faced many challenges primarily protecting and preserving their population’s health. The World Health Organization issued several proposals for communities to better manage and contain the virus ( 1 ). Morocco was no exception to the rule ( 2 ) and decreed drastic emergency measures. Many changes happened on the daily basis for Moroccans like a three-month lockdown, social distancing and sanitation measures, outing or travel restrictions ( 3 ). The balance of the socio-environmental and health system present before the crisis were severely disrupted in many areas ( 4 ). Vulnerable people are particularly sensitive to changes that have emerged since the beginning of the pandemic. There are strong social and health inequities among them and in comparison to the general population ( 5 ). Medical students belong to an at-risk group ( 6 ) and variables such as having a low income ( 7 ), and social distancing from relatives or being under a high stress level ( 8 ) can disturb their overall well-being. Also, they often represent a health resource person for their entourage which can add a significant psychosocial burden ( 9 ). This pandemic disturbed the training methods for aspiring doctors, too. Not only the universities and the student housings did close but the internships were also canceled. Medical students had only online courses ( 10 ). These new experiences could be challenging for them. The main hypothesis of the study was that there are multidimensional psychosocial barriers to have good health, which undermine medical students’ well-being in Marrakesh ( 11 ). We supposed that there are inappropriate coping strategies among them to cope with difficulties encountered in their new daily activities, especially for the new online digital education methods, the change in social interactions and their role as healthcare professionals in the context of the pandemic. The objective of this study was to estimate the well-being level of the pre-graduate medical students in Marrakesh since the onset of the health crisis due to COVID-19 during 2021, and identify their needs and expectations for having a better psychosocial well-being. Also, we wanted to learn their coping strategies to cope with their daily challenges.

## Methods

We conducted a one-phase mixed method study, including a quantitative and a qualitative part from September 2020 to June 2021 in Marrakesh, Morocco, at the Faculty of Medicine and Pharmacy of Marrakesh (FMPM), a public medical university. We conducted the two parts at the same time with specified data collections and analyses to each perspective. After that, we compared and contrasted the two parts with each other based on a triangulation design underlined by a convergence model. So, we combined the findings to obtain the final results.

More precisely, the quantitative part was a cross-sectional study. We did a self-selection sampling which is a non probabilistic sampling method from the 2-first cycles of the medical university curriculum. The inclusion criteria were age≥ 18 years old, mastering of the French language (the higher education’s official language in Morocco), being registered at the FMPM during 2020-2021. A non-inclusion criterion was not to be part of the medical students of the faculty. With this sampling method, we wanted to include as many participants as possible, taking into account our inclusion and non-inclusion criteria. 

An online self-administered questionnaire was elaborated with four main sections. The first one was about the personal characteristics of the students. It included 5 single choice questions to know their gender, age, education level, marital and socioeconomic status, and origin. If the students weren’t Moroccan, they had to write the name of their home country. The second section covered the topic of the changes in the teaching methods to estimate the impact of the educational changes on the well-being. This part had 8 questions including single choice questions and Likert scales questions to evaluate the quality and satisfaction of the online education ranging from 0 (very bad) to 10 (excellent). The third part had 6 questions related to the well-being of the students. The 2 first questions focused on evaluating the students’ perceived well-being. We used Likert scales for estimating the well-being before the onset of the pandemic and one year after the lockdown episode. The Likert scales had ranges from 0 (very low state of well-being) to 10 (complete state of well-being). The participants chose the number which suited them the best to define their perceived well-being so that we could have the mean degree of well-being before the lockdown and the mean degree of well-being one year after the lockdown according to the students themselves. The other 4 questions were multiple choice questions to investigate factors related to 1) the environment for studies that could be improved at the medical school, 2) the need of individual skills which could improve the students’ well-being, 3) the community measures that could foster the well-being at the FMPM, and 4) the health services which could be improved at the FMPM. We based our questions on the literature review and particularly on the four actions areas of the Ottawa Charter that are the strength of the community actions, the development of personal skills, the creation of supportive environments and the reorientation of health services. Finally, as a fourth part we used the Brief-COPE questionnaire (Coping Orientation to Problems Experienced) to measure the different ways to cope with a specific stressful life event (12); in our case it was the coping styles adopted by the medical students since the advent of the pandemic. The Brief-COPE has 14 scales which assess the coping strategies utilized by people. The answers are in a four-point Likert scale ranging from 1 to 4 (I haven’t been doing this at all-sometimes-often-all the time). A higher score means a great utilization of a specific coping style. The reliability and validity of the Brief-Cope were established during the initial development of the tool. The reliability of this questionnaire was examined in our study using Cronbach’s coefficient alpha. The alpha coefficient of the questionnaire was 0.773. This is considered as an acceptable level ( 12 ).

Our questionnaire was sent to the students via the digital learning platform of the FMPM. 

The data were analyzed using SPPS software (Statistical Package for Social Science) version 16.0. The quantitative variables were presented as mean and standard deviation, the categorical variables as numbers and percentages. We used the Fisher's exact test to compare the percentages, and the student's t test to compare two means. The alpha risk significance was set at 5%. 

The qualitative part was a case-study type research with a snowball sampling which is a non-probabilistic sampling method. The population was the same as for the quantitative phase with the same inclusion and non-inclusion criteria.

We conducted semi-structured interviews using an interview guide with four themes on the basis of the literature; 1) the perceived students’ well-being since the COVID-19 pandemic and their main health problems linked to the virus and their effects on the everyday life, 2) their point of view about the new distance learning system: its influence, either positive or negative, on their well-being, 3) difficulties encountered as student doctors and how they could unbalance their overall health, and 4) the adaptation of new habits and their coping strategies. The socio-demographic characteristics of the participants were asked at the end of the interviews.

We recorded and transcribed the interviews. Then, we did a thematic content data and a non-verbal analysis. They were done manually based on a hypotethico-deductive approach.

Then, we did our triangulation analysis using a comparison grid. Indeed, after having our results for each perspective, we classified our main findings in different themes and we compared them to find differences or similarities. 

### Ethical Consideration

The study protocol had a favorable consent from the Marrakesh University Hospital Ethics Committee (reference number 16/2021). This study was conducted according to the guidelines of the Declaration of Helsinki. The objectives of the study were explained to the participants and voluntary informed consent was obtained from all students. All information collected from the participants were kept confidential.

## Results

### Quantitative perspective

#### Description of the participants

The participation rate was 16.6%, with a total of 355 responses from 2138 students. The female to male ratio of the respondents was 1.8. The average age, for the females, was 19.2±1.6 years old and, for the males, it was 19.2±1.8 years old. The proportion of foreign students was 7.9% and students from Marrakech-Safi came first as origin of the students (71.0%) (Table 1).

**Table 1:** Participants’ socio-demographic characteristics

		Participants (n=355)	
		Number (n)	Percentage (%)
Gender	Female	232	65.4
	Male	123	34.6
Study level	1^st^ year	204	57.5
	2^nd^ year	61	17.2
	3^rd^ year	22	6.2
	4^th^ year	32	9.0
	5^th^ year	36	10.1
Origin	Morocco	327	92.1
	Outside Morocco	28	7.9

### Well-being description and coping strategies

**Figure 1:**
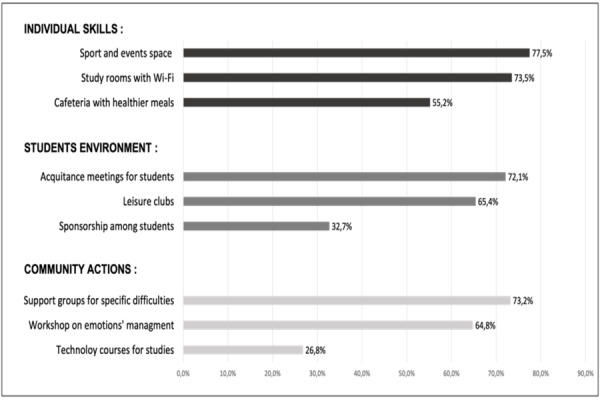
Needs of the medical students regarding health promotion actions

Since the COVID-19 pandemic, the average well-being assessed on a scale of 0 to 10 was 5.4±2.5; only 23.8% of students who had perceived health problems did go to see a healthcare practitioner; 80.6% advocated easy access to psychologists regarding services that promote health within the FMPM. The students brought out three different kinds of factors that could promote their well-being: community actions for medical students, development of personal skills for health and a supportive environment (Figure 1).

According to the Brief-COPE questionnaire, the principal functional coping strategies adopted were the acceptation (5.8±1.7) and planning (4.8±1.6). The main dysfunctional responses were self-distraction (5.0±1.3) and self-blame (4.4±1.5). The religion (5.4±1.8) was another coping strategy largely adopted; it is neither an approach nor an avoidance one (Table 2). 

**Table 2:** Coping strategies adopted by the medical students of the Faculty of Medicine and Pharmacy of Marrakesh according to the Brief-COPE

		Brief-COPE score (n=355)	
		Mean	SD*
Avoidant	Self-distraction	5.0	1.3
	Deny	2.9	1.3
	Substance use	2.3	0.9
	Behavioural disengagement	3.7	1.5
	Self-blame	4.4	1.5
	Venting	4.2	1.6
Approach	Active coping	4.3	1.5
	Emotional support	4.2	1.8
	Use of informational support	4.2	1.8
	Planning	4.8	1.6
	Acceptance	5.8	1.7
Other	Religion	5.4	1.8

*SD: Standard Deviation

### Factors influencing the well-being

Before the pandemic, being a 1st cycle medical student was associated with a better well-being state compared to the 2nd cycle (p<0.001). The change of the well-being degree between before and after was significantly negative (p<0.001). The mean score on a scale from 0 to 10 was estimated at 7.8±2.1 by the students before the pandemic and went down to 5.4±2.5 since the pandemic (p<0.001). We used the student's t test to compare these two means with alpha risk significance sat at 5%. The majority of the students felt a deterioration of their well-being (72.1%) in the post pandemic period while less than a quarter felt an improvement (11.0%) and others had a stable state (16.9%). Also, the well-being state was significantly higher for men than for women (p=0.02) (Table 3).

**Table 3:** The medical students of the Faculty of Medicine and Pharmacy of Marrakesh’s state of well-being before and since the COVID-19 pandemic

		Before the pandemic, how did you estimate your state of well-being?		p*	Since the pandemic, how do you estimate your state of well-being?		p*
		Means	SD**		Means	SD**	
Gender	Female	7.69	2.02	0.470	5.20	2.40	0.020
	Male	7.86	2.34	5.85	2.63		
Study cycle	1^st^ cycle	8.12	1.89	<0.001	5.33	2.49	0.242
	2^nd^ cycle	6.66	2.42	5.69	2.51		
Origin	Morocco	7.76	2.14	0.855	5.38	2.48	0.233
	Outside of Morocco	7.68	2.09	5.96	2.67		

*p: statistical signification using the student's t test; **SD: Standard Deviation

### Qualitative perspective

We conducted 16 interviews, including 2 online, with an equal number of females and males. Nearly half of them were in the 1st cycle of studies (7/16). Three participants were from Sub-Saharian Africa.

### Changes and facing the adversity

The main changes were linked with the routine breaking. First of all, a strict lockdown disrupted the habits of the students, especially because they couldn’t go to university and internships anymore. This marked a phase of great contentment which did not last. Difficulties quickly emerged: it was impossible to have a satisfying social life with a low possibility of having leisure. Isolation and lack of contact with peers plus spending all the time locked up with the same people was very burdensome and potentially a source of conflict within the household. Some of these problems have generally been resolved at the end of the stay-at-home order while some lasted because the state of health emergency was still on. The online education and the establishment of a night curfew represented a source of difficulties for some students. 

*"I used to plan everything after 8 p.m. because I prefer to work at night. I prefer to go out at night. So now I'm deprived of all the daily activities.”*

Nevertheless, 3 out of the 16 students declared having a higher well-being one year after the start of the crisis; they had more free and rest time because of the distance learning as well as greater flexibility in their planning. 

*"For me it was more beneficial to have my education in hand rather to go at fixed schedule and undergo the rhythm, the timeline that the faculty planned.”*

**Figure 2:**
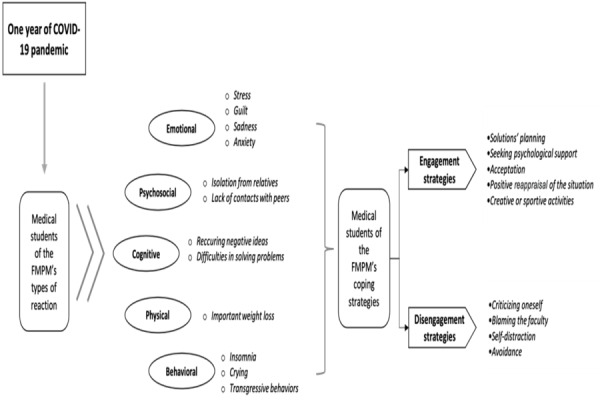
Main reactions and coping strategies of the medical students one year after the onset of the COVID-19 pandemic

Those who had barriers in socializing with others were relieved by the closure of the faculty and satisfied to continue their studies. Their well-being level greatly increased as the crisis broke a routine that previously had negative effects like feelings of depression, burn-out or anxiety. Regarding the students wishing to attend face-to-face classes, they remained greatly dissatisfied. Almost half of the participants (7/16) declared that they had developed psychosocial disorders. A few sought medical or psychological support: two called the listening unit, and one saw a psychologist. To face the adversity the students put in place coping strategies. A minority had coping avoidance strategies including the expression of negative emotions towards the crisis situation without adopting functional behavior over the long term. The majority (11/16) said they had put in place engagement-type strategies. Only three students with active adaptation strategies declared a feeling of well-being that was satisfying or even greater than before the pandemic, even if it represented a challenge at the psychosocial level at a moment or another time (Figure 2).

*“It’s been over a year now. I’ve adjusted and it has become my lifestyle.”*

### Medical students’ needs to improve their psycho-social well-being

There were two categories of needs. Firstly, the needs of the acquisition of psychosocial skills were connected with the management of negative emotions and especially stress since they all had perceived a feeling of intense stress at some point during the crisis. 

*“I realized that it was me who was under stress! So, I could control my stress. It's just that I didn't have the method.”*

Time management for e-learning represented a challenge for half of students. Having educational sessions to strengthen autonomy and the quality of personal work provided seemed important for them. The main health-related needs were benefiting from psychological support by a health professional and having a space to express their emotions to free themselves from psychological burdens. All said they knew the existence of the listening unit within the faculty but most said they did not know its action modes which prevented them from calling it for help. However, the ones who had already benefited from this unit were largely satisfied, whether they had asked for a psychological assistance, a request for information and guidance regarding the new coronavirus or help in relation to their internships.

*“With the listening unit I felt calm and reassured. “*

## Discussion

The FMPM, in addition to training future doctors, provides continuing education for students and teachers. Also, a cafeteria is available as well as a library with a reading room. There are spaces with free Wi-Fi zones. A listening unit was created to provide psychosocial support to students in difficulty and there are clubs and students’ associations which do charitable work for the local community.

### Triangulation findings

The triangulation analysis showed a logical concordance between the two perspectives of this study as we obtained similar and complementary data. This design permitted to directly compare and contrast quantitative results with qualitative findings and to expand the quantitative results.

Generally, navigating the changes over more than a year after the beginning of the COVID-19 pandemic led to an overall decrease of the medical students’ well-being. They adopted various reactions and coping strategies which impacted their psychosocial well-being sometimes for good and sometimes not. Acceptation and planning were the main coping strategies adopted. These types of response, according to the literature review, are more likely associated with a low perceived stress. On the contrary, the avoidant coping strategies such as the expression of negative emotions were related to a high-perceived stress level and a greater psychological distress. The main difficulties of the students were social isolation and problems to adapt to distance learning. The ones who had most barriers were the 1st cycle students. We can differentiate two main types of coping: avoidance strategies that involve rather passive and potentially negative long-term behaviors regarding the general state of well-being of a person, engagement strategies that involve more active attitudes to problem solving. The students with a positive experience and a feeling to grow were the ones who mostly had a good capacity of resilience in adopting engagement-type coping strategies ( 12 ).

Globally, there was a decrease of the students’ well-being after the onset of the pandemic as it has been found in the other regions of the world ( 13 , 14 ). More precisely, medical students from developing Muslim countries like Morocco such as Jordan ( 15 ) and Bangladesh ( 16 ) had also their well-being negatively affected as it was the case in our study. 

### Factors associated to well-being

The well-being degree of medical students was associated to factors like gender, psychosocial support and logistical uncertainty related to studies (exams planning, postponing contests). We found the same elements in France ( 8 ), Saudi Arabia ( 17 ) and Switzerland ( 18 ).

### Religion as a coping strategy

Religion was one of the main coping strategies adopted by the students according to the Brief-COPE. It represents neither a strategy of engagement nor an avoidance one in this questionnaire, but it differs according to the contexts. A study done in the UK informs that religion would impact the overall health either directly or indirectly as long as it is linked to beliefs and world views ( 19 ). This strategy is seen as a central mechanism for dealing with particularly stressful situations ( 20 ).

### Axes of recommendations

The impact of the study is to optimize the well-being of the medical students in Marrakesh in a post-pandemic future in giving several realistic and easy doable recommendations: 1) There are three important components to focus on according to our general results to improve the medical students’ well-being as a community: improving a supportive learning environment, strengthening community actions for students and developing personal skills useful for the medical studies and practice. These axes need to be developed even though initial actions are already put in place. To improve resilience in time of major changes it seems central to boost the capacity of the students by giving them enough resources that sustain their psycho-social well-being ( 18 ). Their skills in terms of adaptability to their environment should be strengthened ( 21 ) to achieve a better well-being state in their everyday life after a period of strong restrictions. Before the pandemic, some countries like China, the UK or the USA created health promoting universities within the Ottawa Charter in order to foster the well-being of the students ( 22 , 23 ), 2) An appreciative inquiry to overcome adversity in bringing people together and increase students’ engagement with the help of mental health professionals could be a favorable way to input positive changes. 3) Any actions put in place to improve the students ‘well-being at the FMPM should be done in a salutogenesis perspective to put efforts on supportive factors and implement innovative psychosocial changes. This concept takes its origin in the health psychology field. It is about focusing on the protective factors, which could improve the well-being rather than what causes health problems ( 24 , 25 ). 

### Limitations and strengths

A first limitation was that the survey took place during the state of emergency: the social distancing measures and the closure of the faculty induced barriers to access to the students. A second limitation was that we did not have a representative sample. Furthermore, the study was done among pre-graduate medical students at the public university and cannot represent the students from the private medical university of Marrakesh as they have different socioeconomic determinants. Another limitation about the samples is that we had a few volunteers for the interviews of the qualitative part.

On the other hand, the main strength of the study is its mixed design which allows exploring our subject from several perspectives so that the qualitative part made possible to better understand the quantitative results which strengthened our findings. In addition to this, we had a great enthusiasm for the online questionnaire with a participant rate of 16.6%, which is a good percentage regarding the specific context of the COVID-19 pandemic and doing this part of the survey online. 

 This study gives information to develop a better education environment at the FMPM. It shows what is interesting to focus on to improve the well-being of the medical students before and after a crisis period ( 26 ). Furthermore, this study provides a basis for long-term planning to have a healthier medical school in Marrakesh. So, it should help prioritize the decision-making and the management of effective health promotion actions as it is done in many universities across the world including countries like Canada or New Zealand ( 27 ).

## Conclusion

Maintaining a good well-being state for medical students of Marrakesh, Morocco, as for the rest of the world, has been a challenge since the onset of the COVID-19 pandemic. To cope with this special situation, medical schools should support the students and encourage psychological and socio-environmental supports via, for instance, the promotion of student clubs or the development of a sponsorship program to foster the communication between peers. Indeed, it seems essential to strengthen community actions for students. Concerning the development of personal skills useful for studies, they appear to be the main components to focus on to promote medical students’ well-being at the FMPM. This means solution-based actions such as workshops on how to manage stress or how to be well-organized for distance learning. In short, actions need to be taken at the FMPM in order to foster the overall well-being of the medical students of Marrakesh based on a socioecological approach.

## Acknowledgement

Thanks are due to all participating students, the heads of the FMPM and its logistics team as well as Dr. Adil Mansouri for his recommendations.

**Conflict of Interest:** None declared.
